# Long-Lasting Hippocampal Synaptic Protein Loss in a Mouse Model of Posttraumatic Stress Disorder

**DOI:** 10.1371/journal.pone.0042603

**Published:** 2012-08-10

**Authors:** Leonie Herrmann, Irina A. Ionescu, Kathrin Henes, Yulia Golub, Nancy Xin Ru Wang, Dominik R. Buell, Florian Holsboer, Carsten T. Wotjak, Ulrike Schmidt

**Affiliations:** 1 Max Planck Institute of Psychiatry, Munich, Germany; 2 Department of Child and Adolescent Mental Health, University Clinic Erlangen, Erlangen, Germany; University of Alabama at Birmingham, United States of Ameica

## Abstract

Despite intensive research efforts, the molecular pathogenesis of posttraumatic stress disorder (PTSD) and especially of the hippocampal volume loss found in the majority of patients suffering from this anxiety disease still remains elusive. We demonstrated before that trauma-induced hippocampal shrinkage can also be observed in mice exhibiting a PTSD-like syndrome. Aiming to decipher the molecular correlates of these trans-species posttraumatic hippocampal alterations, we compared the expression levels of a set of neurostructural marker proteins between traumatized and control mice at different time points after their subjection to either an electric footshock or mock treatment which was followed by stressful re-exposure in several experimental groups. To our knowledge, this is the first systematic *in vivo* study analyzing the long-term neuromolecular sequelae of acute traumatic stress combined with re-exposure. We show here that a PTSD-like syndrome in mice is accompanied by a long-lasting reduction of hippocampal synaptic proteins which interestingly correlates with the strength of the generalized and conditioned fear response but not with the intensity of hyperarousal symptoms. Furthermore, we demonstrate that treatment with the serotonin reuptake inhibitor (SSRI) fluoxetine is able to counteract both the PTSD-like syndrome and the posttraumatic synaptic protein loss. Taken together, this study demonstrates for the first time that a loss of hippocampal synaptic proteins is associated with a PTSD-like syndrome in mice. Further studies will have to reveal whether these findings are transferable to PTSD patients.

## Introduction

Posttraumatic stress disorder (PTSD) is a debilitating psychiatric disease occurring in the aftermath of traumatic events like natural disasters, combat or sexual abuse. PTSD core symptoms comprise persistent re-experiencing of traumatic hotspots, avoidance of trauma-associated stimuli, emotional numbing and nervous hyperarousal. Although clinical and animal studies already identified PTSD candidate molecules like FK506 binding protein 5 (FKBP5) [1] and cyclin-dependent kinase 5 (CDK5) [2] and revealed gene x environment interactions [3]–[5] as well as alterations of the HPA axis [6] and sympathetic nervous system overdrive [7] to contribute to PTSD pathogenesis, the molecular pathomechanisms underlying this anxiety disorder still remain elusive. Since PTSD usually does not appear until four weeks after the traumatic experience and moreover can pass into a chronic form [8], deciphering the *long-term* molecular sequelae of traumatic stressors is central to unraveling PTSD pathogenesis. Surprisingly, there are only few reports on this topic and we found no systematic study examining the long-term effects of acute traumatic stress on the expression of neurostructural markers *in vivo*.

Besides molecular changes, the majority of PTSD patients exhibit volume changes of certain brain regions, most notably a volume loss of the hippocampus [9], [10]. We demonstrated before that trauma-induced hippocampal shrinkage can also be observed in mice developing a PTSD-like syndrome in the aftermath of a single traumatic footshock [11].

With the aim to further elucidate the hitherto largely unkown long-term molecular changes underlying this trans-species posttraumatic hippocampal volume loss, we compared the expression levels of a set of central nervous system (CNS) plasticity markers between mice exposed to a traumatic footshock leading to a PTSD-like syndrome and littermates subjected to mock treatment. For two different purposes, namely for assessment of PTSD-like symptoms and for re-exposure of mice to trauma-related environmental cues, we subjected several groups of mice to a behavioral test battery 28 days after application of traumatic stress. Expression analyses were performed employing tissue lysates prepared from bilateral murine hippocampi at different time-points after shock or control treatment. We examined glial fibrillary acidic protein (GFAP), microtubule associated protein 2 (MAP-2), and neurofilament H as glial, dendritic, and neuronal/axonal markers, respectively. In addition, we selected and analyzed homer 1b/c as postsynaptic-, and the two synaptic vesicle (SV) proteins synapsin and synaptophysin as presynaptic marker proteins.

Both synapsin and synaptophysin have hitherto not been linked to animal or human PTSD but to schizophrenia and bipolar disorder [12]–[14]. Several studies in rats and a few studies in mice and marmosets revealed cerebral expression levels of these two SV proteins to be markedly influenced by chronic prenatal, chronic early life, and chronic adult stress [15]–[23]. These reports indicate that type, duration, and time of onset of the chronic stressor as well as gender and species of the affected individual differentially modulate the impact of chronic stress on cerebral synapsin and synaptophysin levels thereby stressing the complexity of SV protein regulation. In contrast, the effects of *acute* stress and/or re-exposure to trauma related environmental cues on the expression of synapsin and synaptophysin have been studied less intensively, but it already has become clear that chronic and acute stressors differentially impact on SV protein expression levels [18], [19], [24]. Information on the influence of stress on CNS expression of the other candidate proteins tested here is sparse and has hitherto been mainly analyzed in chronic stress paradigms [25]–[29].

Taken together, we are the first to demonstrate a marked long-term reduction of synaptic proteins in the hippocampus of mice exhibiting PTSD-like symptoms. In addition, we demonstrate that the SSRI antidepressant fluoxetine, which was reported before to effectively alleviate the PTSD-like syndrome in mice [30] and PTSD symptoms in humans [31], counteracts trauma-induced hippocampal synaptic protein loss.

## Results

### A PTSD-like Syndrome in Mice is Accompanied by a Long-term Reduction of Hippocampal Synaptic Proteins

**Figure 1:**
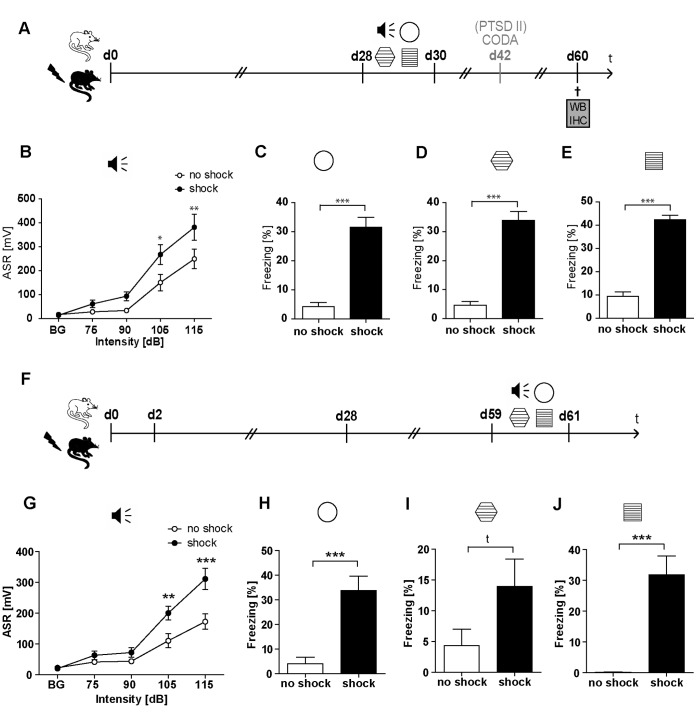
Traumatic footshock induces a long-lasting PTSD-like behavioral syndrome in mice. C57BL/6NCrl mice were either subjected to a single electric footshock (“shock”) or, mock treatment (“no shock”). Mice of batch PTSD I were subjected to behavioral testing on days 28–30 (**A–E**), while mice of batch PTSD III were behaviourally tested on days 59–61; (**F–J**): the intensity of the acoustic startle reflex (ASR) was analyzed in response to white noise pulses of 50 dB (background, BG) and 75, 90, 105, and 115 dB (**B,G**). The generalized fear response was assessed by evaluating the freezing response both in a neutral context (**C, H**) and in a grid context similar to the shock chamber (**D, I**). The conditioned fear response was analyzed by evaluation of the freezing response in the shock context (**E, J**); freezing duration was normalized to the 3 min observation intervals (Freezing [%]). (**A–E**) Presented data are means ± SEM, n = 16 (batch PTSD I, behavioral testing d28–30). Statistical analysis was performed using students *t*-test (neutral context d28–30: t (29) = 7.442, p<0.001; grid context d28–30: t (28) = 8.582, p<0.001; shock context d28–30: t (29) = 12.36, p<0.001; C–E) and, in case of the acoustic startle response (ASR), using two-way repeated measures ANOVA and Bonferroni post-hoc tests (Acoustic startle response d28–30: F_1,29 shock = _6.39; p = 0.017; F_4,116 INT x shock_ = 2.91; p = 0.024; B) and is indicated by * p<0.05, ** p<0.01, *** p<0.001. (**F–J**) Presented data are means ± SEM, n = 14 (no shock), n = 16 (shock) (batch PTSD III, behavioural testing d59–61). Statistical analysis was performed using students *t*-test (neutral context d59–61: t (28) = 4.423, p<0.001; grid context d59–61: t (28) = 1.787, p = 0.085; shock context d59–61: t (23) = 4.868, p<0.001; H–I) and, in case of the acoustic startle response (ASR), using two-way repeated measures ANOVA and Bonferroni post-hoc tests (Acoustic startle response d59–61: F_1,28 shock_ = 21.490; p<0.001; F_4,140 INT x shock_ = 4.710; p = 0.001; G) and is indicated by t p<0.1, ** p<0.01, *** p<0.001.

For all analyses described here, we employed our previously published PTSD mouse model [30]. We started with analyzing the time course of the expression levels of the SV protein synapsin. Different groups of mice were sacrificed 2, 28, and 60 days after exposure to either traumatic footshock or mock treatment. Mice sacrificed on day 60 were subjected to a behavioral test battery on days 28–30 after footshock application to assess behavioral symptoms. To evaluate behavioral alterations in the *long term*, an additional batch of mice was behaviorally tested on day 60 after footshock (Figure 1F–J). Behavioral analyses included re-exposure stress since mice were subjected to trauma related cues upon analysis of their conditioned fear response. After mice had been sacrificed, tissue lysates of bilateral hippocampi (HC), prefrontal cortices (PFC), and cerebella (CER) were prepared for analysis of the expression levels of a set of neurostructural marker proteins.

As published previously [30], on day 28, footshocked mice exhibited a significantly higher expression of conditioned fear, generalized fear and hyperarousal compared to mock treated control mice (Figure 1B–E). In addition, we show here for the first time that this behavioral PTSD-like syndrome persists at least till day 60 (Figure 1 F–J). Mice sacrificed on day 2 or day 28 were *not* subjected to behavioral analyses at all. In a previous study employing the same PTSD mouse model, we demonstrated that on day 1 after footshock traumatized mice show a significantly increased conditioned fear response which persists at least till day 28, while their generalized fear response increases over time [30].

**Figure 2:**
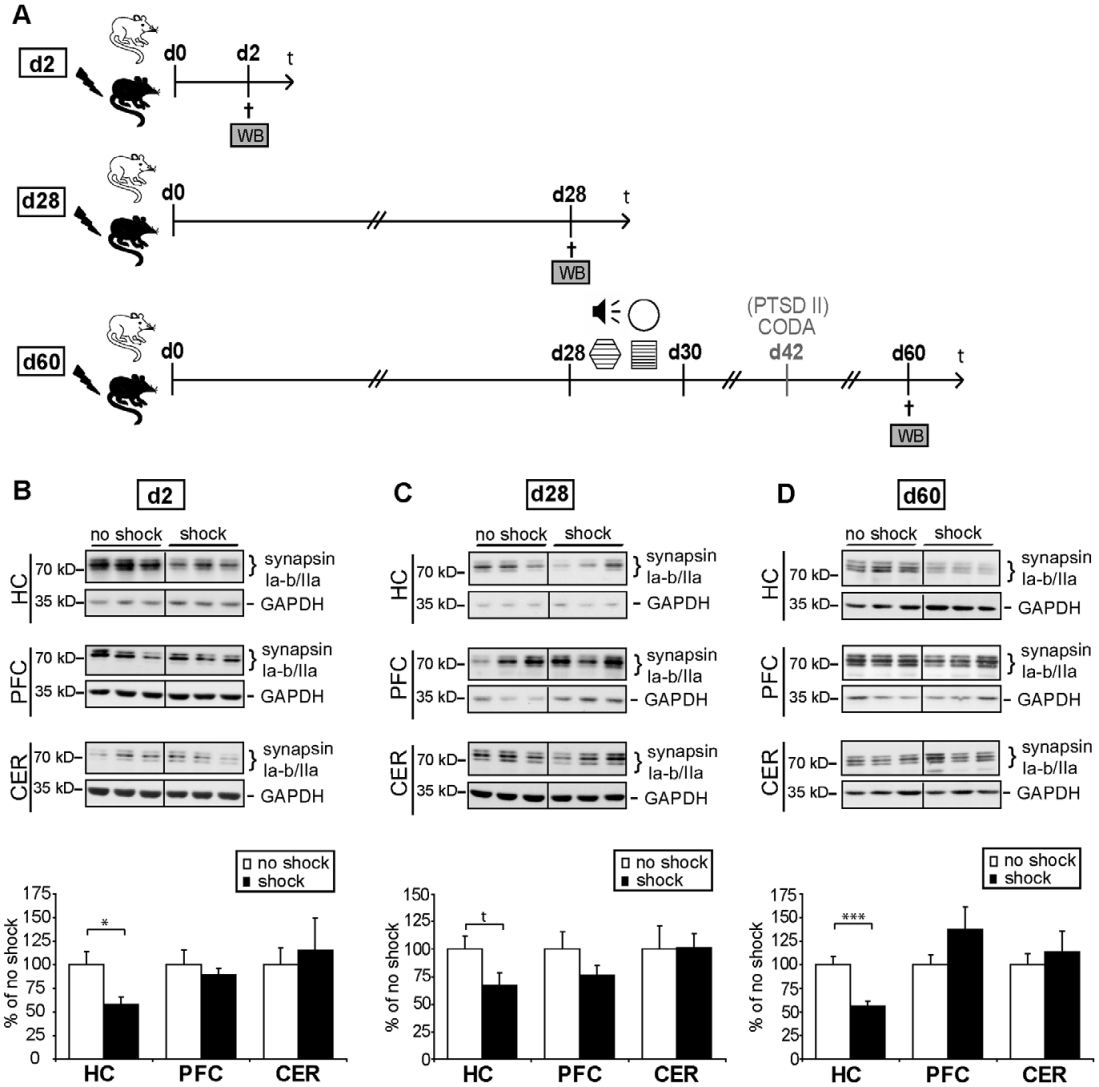
Acute traumatic stress in combination with re-exposure stress leads to a long-lasting decrease of hippocampal synapsin Ia-b/IIa expression. (**A**) Course of experiment: C57BL/6 NCrl mice (batch PTSD I and II, see Table 2) were subjected to a single electric footshock, (“shock”) or mock treatment (“no shock”). Different groups of them were sacrificed at different time-points after footshock application. Three brain regions were dissected and tissues were lysed and subjected to western blot analysis (WB) for determination of synapsin expression levels. On day 28 after exposure to footshock or mock treatment, mice were subjected to behavioral tests (which included re-exposure to the trauma context) and sacrificed on day 60, while mice sacrificed on day 2 or day 28 were *not* subjected to behavioral analyses. For assessment of PTSD-like symptoms, the acoustic startle response, the generalized fear response (both in a neutral context and in an experimental context similar to the shock context) and the conditioned fear response were assessed as described in the methods section and in Figure 1. Mice of batch PTSD II were additionally subjected to a CODA test on day 42 (described in the methods section and in Figure S2). Figure S1 provides further details of behavioral protocols. (**B–D**) Representative immunoblots show expression levels of synapsin Ia-b/IIa and, for control, glycerinaldehyd-3-phosphat-dehydrogenase (GAPDH) of footshocked (“shock”) or control (“no shock”) mice, respectively. Grouped graphical inserts belong to one and the same gel. Graphs show expression levels of synapsin Ia-b/IIa after normalization to GAPDH 2 days (**B**), 28 days (**C**), and 60 days (**D**) after shock in lysates of bilateral hippocampi (HC), prefrontal cortices (PFC), or cerebella (CER) of footshocked and control mice, respectively. Synapsin Ia–b/IIa expression levels of footshocked mice are presented as percent of those of control mice (set at 100%). Plotted data represent means ± SEM, n = 6 for d2 and d28, n = 12 (batches PTSD I and PTSD II) for d60. Statistical significance was calculated using students *t*-test and is indicated by t p<0.1, * p<0.05 and *** p<0.001 (d2: HC t (10) = 2.631, p = 0.025, PFC t (10) = 0.603, p = 0.560, CER t (10) = 0.398, p = 0.698; d28: HC t (10) = 1.963, p = 0.078, PFC t (10) = 1.279, p = 0.229, CER t (10) = 0.044, p = 0.965; d60: HC t (22) = 4.332, p<0.001, PFC t (22) = 1.408, p = 0.173, CER t (10) = 0.540, p = 0.601).

We found synapsin Ia–b/IIa protein levels to be significantly reduced in the hippocampus both on day 2 (Figure 2B: HC, t (10) = 2.631, p = 0.025) and day 60 after application of traumatic footshock (Figure 2D: HC, t (22) = 4.332, p<0.001). However, on day 28, i.e. in a batch of mice *not* subjected to any behavioral testing procedure, we detected only a trend in synapsin Ia–b/IIa reduction (Figure 2C: HC, t (10) = 1.963, p = 0.078). The fact that we found not only synapsin but none of the proteins tested here to be significantly altered on day 28 after application of footshock stress, strongly supports our hypothesis that re-exposure to trauma-related environmental cues on days 28–30 probably amplifies and prolongs the molecular stress response in the CNS. As between day 28 and day 60 synapsin reduction persisted without any in-between subjection of mice to further stressors, we regard the observed protein level changes that persisted from re-exposure on day 28 until day 60 as long-lasting alterations. In contrast to the hippocampus, synapsin protein levels remained unchanged in the prefrontal cortex and the cerebellum at all the three time points analyzed (Figure 2B–D, summarized in Table 1.

**Table 1:** Overview of changes in candidate protein expression levels.

	d2	d28	d60	Fluoxetine d74
**synapsin Ia–b/IIa**	↓	↓**^t^**	↓	↑
**synaptophysin**	↓**^t^**	↔	↓	↑
**homer 1b/c**	↔	↔	↓	↔
**neurofilament H**	↓	↔	↓**^t^**	↔
**MAP-2**	↔	↔	↔	↔
**GFAP**	↔	↔	↔	↔

**Figure 3:**
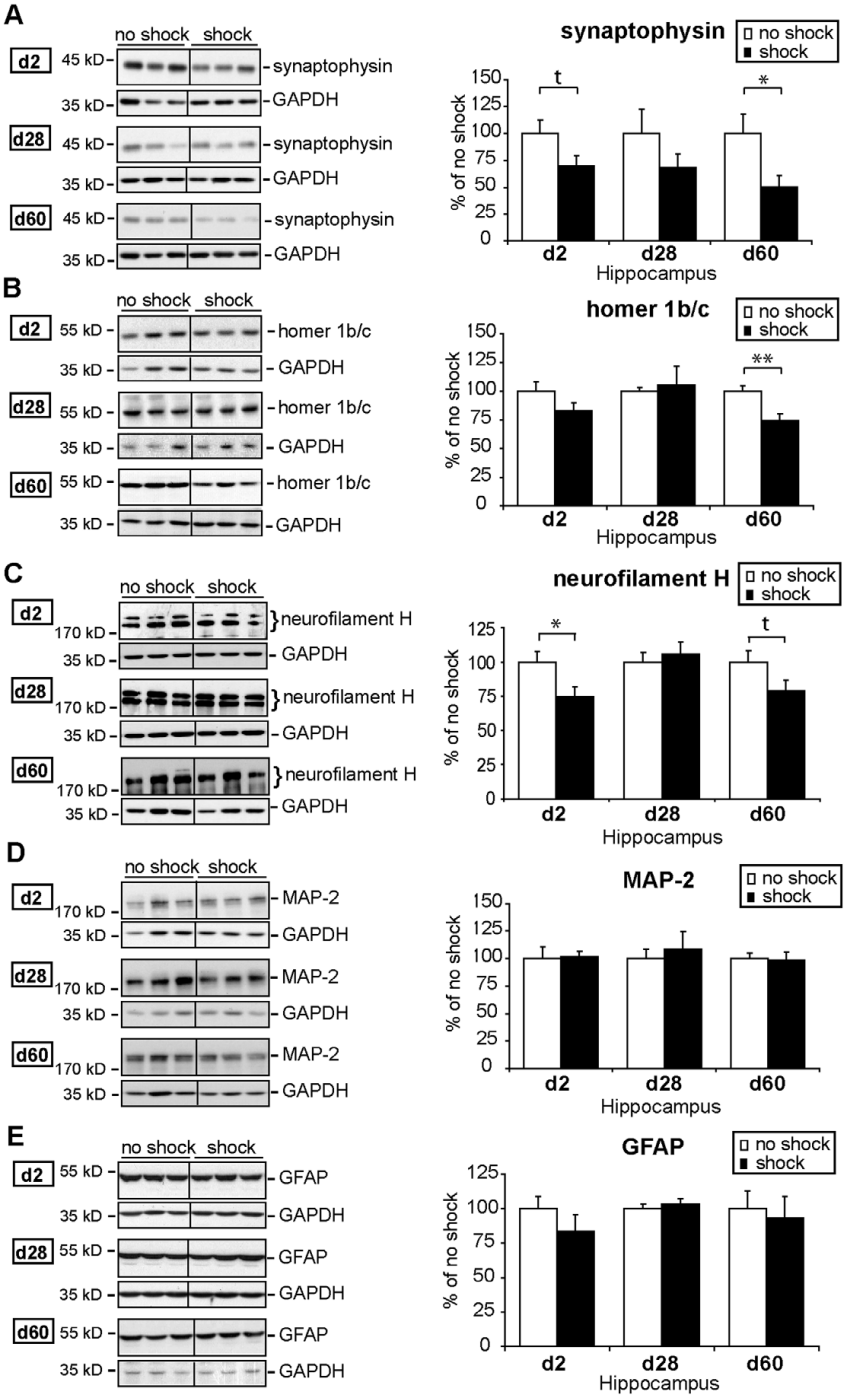
Time course of hippocampal synaptophysin, homer 1b/c, neurofilament H, MAP-2, and GFAP expression after traumatic footshock and re-exposure stress. The course of the experiment (batches PTSD I and II, see Table 2) is identical to that depicted in Figure 2A. Protein expression levels of synaptophysin (**A**), homer 1b/c (**B**), neurofilament H (**C**), microtubule-associated protein 2 (MAP-2) (**D**), and glial fibrillary acidic protein (GFAP) (**E**) were assessed by western blot (WB) in lysates of pooled bilateral hippocampi (HC) of footshocked and control treated mice sacrificed on day 2, 28, or 60 after shock. (**A–E**) Representative immunoblots show expression levels of all candidate proteins mentioned and, for control, of glycerinaldehyd-3-phosphat-dehydrogenase (GAPDH) of footshocked or control (no shock) mice, respectively. Grouped graphical inserts belong to one and the same gel. Graphs show expression levels of candidate proteins after normalization to GAPDH. Candidate protein expression levels assessed in shocked mice are presented as percent to those of non-shocked control mice set at 100% (% of control). Plotted data represent means ± SEM, n = 6 for d2 and d28, n = 12 from two independent batches for d60 (except for synaptophysin for which only one batch, PTSD I, n = 6) was employed. Statistical significance was calculated using students *t*-test and is indicated by t p<0.1 * p<0.05 (synaptophysin: d2: t (10) = 1.869, p = 0.091; d28: t (10) = 1.209, p = 0.254; d60: t (10) = 2.351, p = 0.040; homer 1b/c: d2: t (10) = 1.573, p = 0.147; d28: t (8) = 0.259, p = 0.801; d60: t (22) = 3.552, p = 0.002; neurofilament H: d2: t (10) = 2.422, p = 0.036; d28: t (10) = 0.537, p = 0.603; d60: t (21) = 1.873, p = 0.075; MAP-2: d2: t (10) = 0.155, p = 0.880; d28: t (10) = 0.495, p = 0.631; d60: t (22) = 0.152, p = 0.881; GFAP: d2: t (10) = 1.116, p = 0.291; d28: t (10) = 0.667, p = 0.520; d60: t (22) = 0.339, p = 0.738).

Arrows indicate the relation of candidate protein expression levels in shocked mice to either non-shocked (d2, d28, d60 batches) or untreated (fluoxetine batch) control mice. This is a summary of the data presented in Figures 2 and 3 (batch PTSD I and II) as well as in Figure 6 (batch PTSD IV). Abbreviations: trend (t); hippocampus (HC); microtubule associated protein 2 (MAP-2); glial fibrillary acidic protein (GFAP).

To find out whether this enduring decrease of synapsin (Figure 2) reflects an overall structural alteration of the synapse, we analyzed another synaptic vesicle protein, i.e. synaptophysin, which is widely used as a marker for synaptic density. We found a long-term reduction of hippocampal synaptophysin expression in traumatized mice (Figure 3A): on day 2, hippocampal synaptophysin levels showed at least a trend towards reduction (Figure 3A: t (10) = 1.869, p = 0.091), on day 28 synaptophysin decrease was not significant but quite pronounced on day 60 after shock (Figure 3A: t (10) = 2.351, p = 0.040). Thus, both synapsin Ia–b/IIa and synaptophysin protein levels were downregulated in the hippocampus of C57BL/6 NCrl mice after traumatic footshock suggesting that changes in the total number of synaptic vesicles or even in the total number of synapses might accompany the response of the mouse brain to traumatic stress.

Then, we analyzed the expression levels of homer 1b/c, a postsynaptic marker protein. We found a decrease in hippocampal homer 1b/c expression levels on day 60 after footshock (Figure 3B: t (22) = 3.552, p = 0.001), but neither on day 2 nor on day 28, indicating that the reduction of homer 1b/c expression either occurs with delayed onset or is amplified by the stressful behavioral testing procedure on day 28. These findings, together with the observed decrease in SV proteins, point at a holosynaptic protein loss in the shrinked hippocampus [11] of mice suffering from a PTSD-equivalent syndrome.

Next, to evaluate whether in addition a diminution of neurons or glia cells contribute to the footshock-elicited hippocampal shrinkage, we analyzed the protein expression levels of the pan-neuronal marker neurofilament H, the dendritic marker microtubule-associated protein 2 (MAP-2), and the astroglial marker glial fibrillary acidic protein (GFAP). Neurofilament H expression was slightly reduced on day 2 after footshock (Figure 3C: t (10) = 2.422, p = 0.035), remained unchanged on day 28 but showed a trend towards a reduction on day 60 after footshock (Figure 3C: t (21) = 1.873, p = 0.075), while both MAP-2 and GFAP expression levels were not significantly altered at any of the three time points analyzed (Figure 3D, E, see Table 1 for summary of all tested proteins). Since neurofilaments are enzymatically degraded in synaptic structures [32], it is less likely that the subtle decrease in neurofilament H expression found here reflects synaptic loss, but more likely that it reflects persistent axonal degradation.

**Figure 4:**
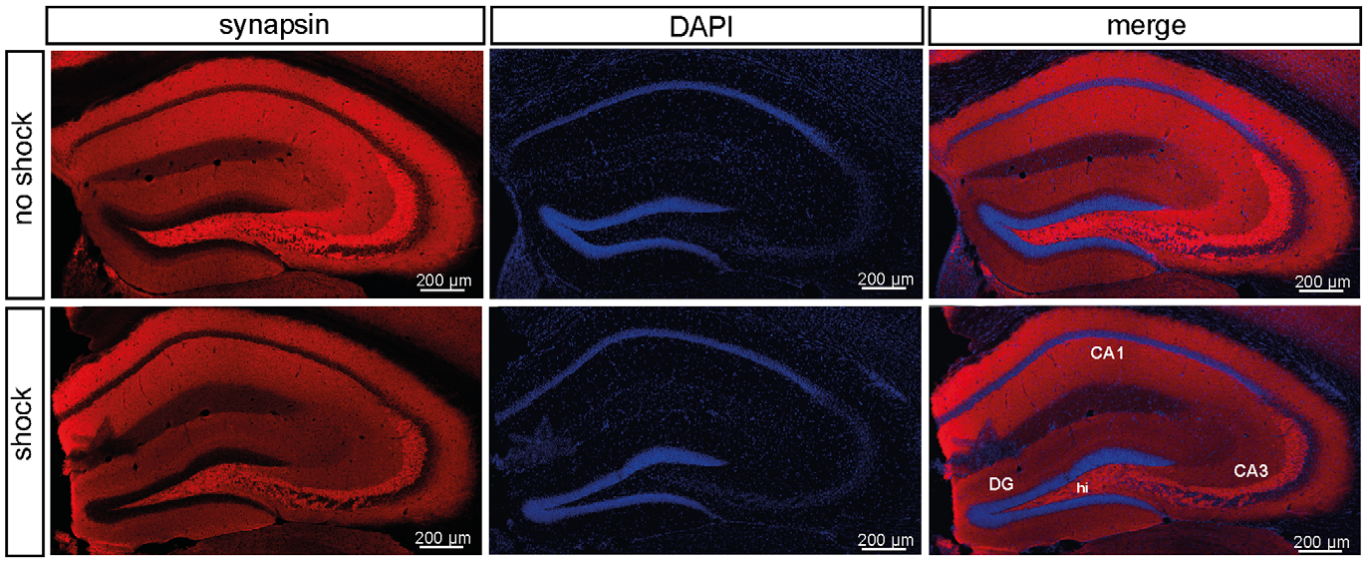
Immunohistochemical analysis of hippocampal synapsin expression. Representative images (from one mouse out of 6 mice per test condition) of coronal hippocampal sections (bregma −1.6 mm) showing the localization of hippocampal synapsin expression in shocked and non-shocked mice sacrificed 60 days after subjection to footshock (batch PTSD II, behavioral testing d28–30) (left panel). Slices were counterstained with DAPI (middle panel). The overlay of the DAPI and anti-synapsin stained sections is presented in the right panel (merge). Abbreviations: Cornu ammonis areas 1 and 3 (CA1, CA3), dentate gyrus (DG), hilus region (hi).

To determine the synapsin expression sites in the murine hippocampus, we performed a immunohistochemical (IHC) analysis of synapsin I/II expression in the hippocampus of traumatized and control mice sacrificed 60 days after footshock (Figure 4). As expected from studies in rats [33], [34] and one study in mice [35], synapsin I/II expression was predominantly localized to the CA3 and the hilus region, while it was sparse in CA1 (Figure 4).

### Hippocampal Synaptic Protein Expression Levels Negatively Correlate with Conditioned and Generalized Fear but not with Hyperarousal

**Figure 5:**
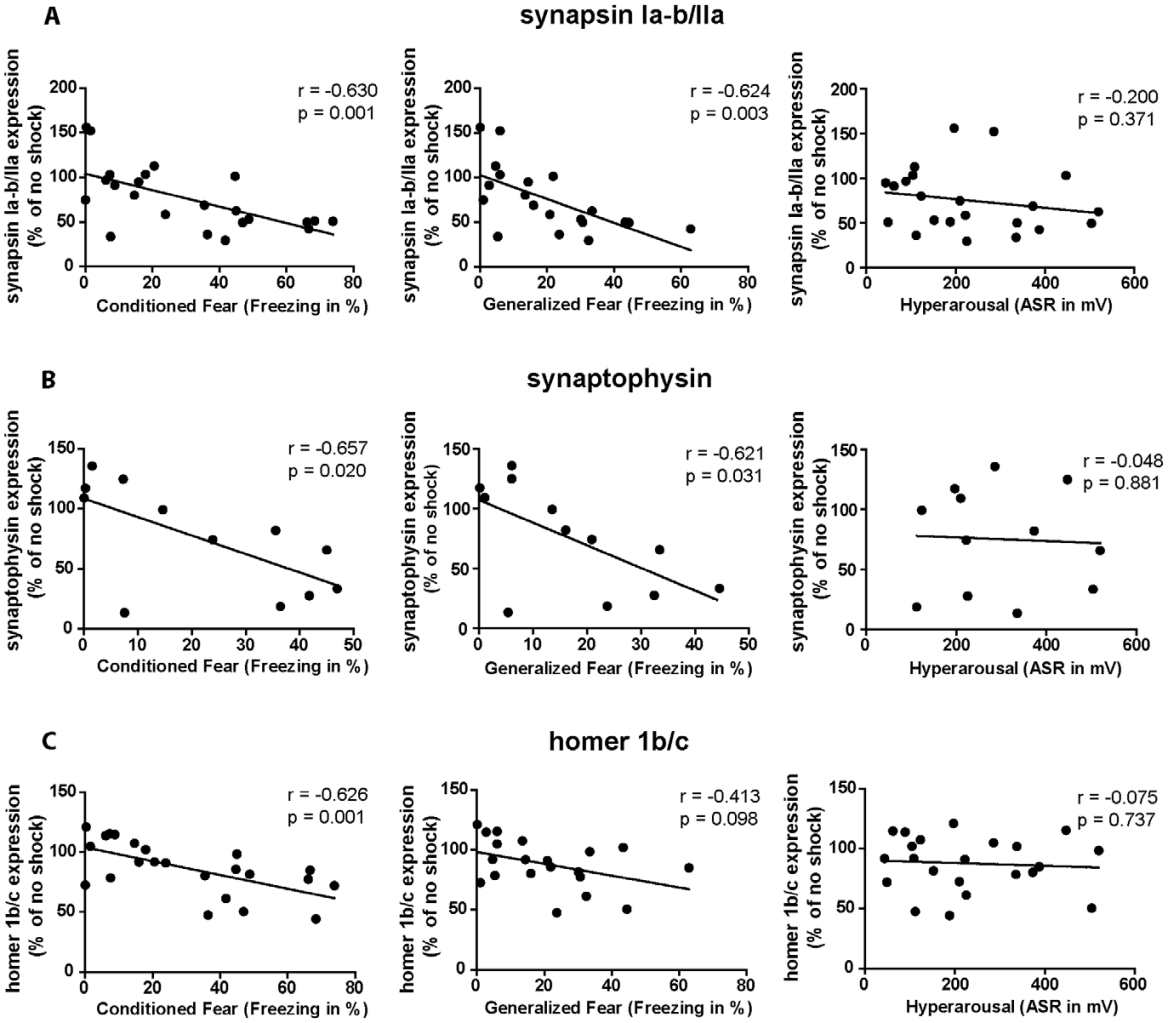
Hippocampal SV protein expression correlates negatively with the generalized and the conditioned fear response but not with hyperarousal. (**A–C**) Statistical correlation of protein expression levels with behavioral data in shocked and non-shocked mice: intensities of the different symptoms of the murine PTSD-like syndrome, i.e. conditioned fear, generalized and the acoustic startle response (ASR), were plotted against the relative day-60 protein expression levels (% of no shock) of synapsin Ia–b/IIa (**A**), synaptophysin (**B**) and homer 1b/c (**C**) of *both* shocked and control mice (i.e. each cloud of dots comprises both shocked and mock-treated mice). Pearson correlation coefficients (“r”) were calculated and a two-tailed student’s *t*-test was performed (“p”), n = 12 (batch PTSD I and II) for synapsin Ia–b/IIa and homer 1b/c, n = 6 (batch PTSD I only) for synaptophysin (synapsin: conditioned fear: r = −0.630, p = 0.001; generalized fear: r = −0.624, p = 0.003; hyperarousal: r = −0.200, p = 0.371; synaptophysin: conditioned fear: r = 0.657, p = 0.020; generalized fear: r = −0.621, p = 0.031; hyperarousal: r = −0.048, p = 0.881; homer 1b/c: conditioned fear: r = −0.626, p = 0.001; generalized fear: r = −0.413, p = 0.098; hyperarousal: r = −0.075, p = 0.737).

Aiming to elucidate the function of the here observed hippocampal synaptic protein loss in the development and maintenance of the PTSD-like syndrome, we statistically correlated the strength of the three different footshock-elicited symptoms assessed on day 28 after footshock or mock treatment (i.e. clusters of dots depicted in figures 5A–C comprise values of both shocked and control mice) with the expression intensities of hippocampal synapsin Ia–b/IIa, synaptophysin, and homer 1b/c assessed on day 60 after footshock or mock treatment. Correlation analyses were performed according to the procedure published by Wu et al. (2007) and Zink et al. (2010) [36], [37]. Our analyses revealed that the expression levels of each of the three synaptic proteins tested correlate negatively with the intensity of conditioned fear (synapsin Ia–b/IIa: Figure 5A: r = −0.630, p = 0.001; synaptophysin: Figure 5B: r = −0.657; p = 0.020; homer 1b/c: Figure 5C: r = −0.626; p = 0.001) as well as with the intensity of generalized fear (synapsin Ia–b/IIa: Figure 5A: r = −0.624, p = 0.003; synaptophysin: Figure 5B: r = −0.621; p = 0.031; homer 1b/c: Figure 5C: r = −0.413; p = 0.098) but interestingly not with the intensity of the startle reflex which mirrors their arousal status.

### Chronic Fluoxetine Treatment Counteracts Trauma-induced SV Protein Reduction

**Figure 6:**
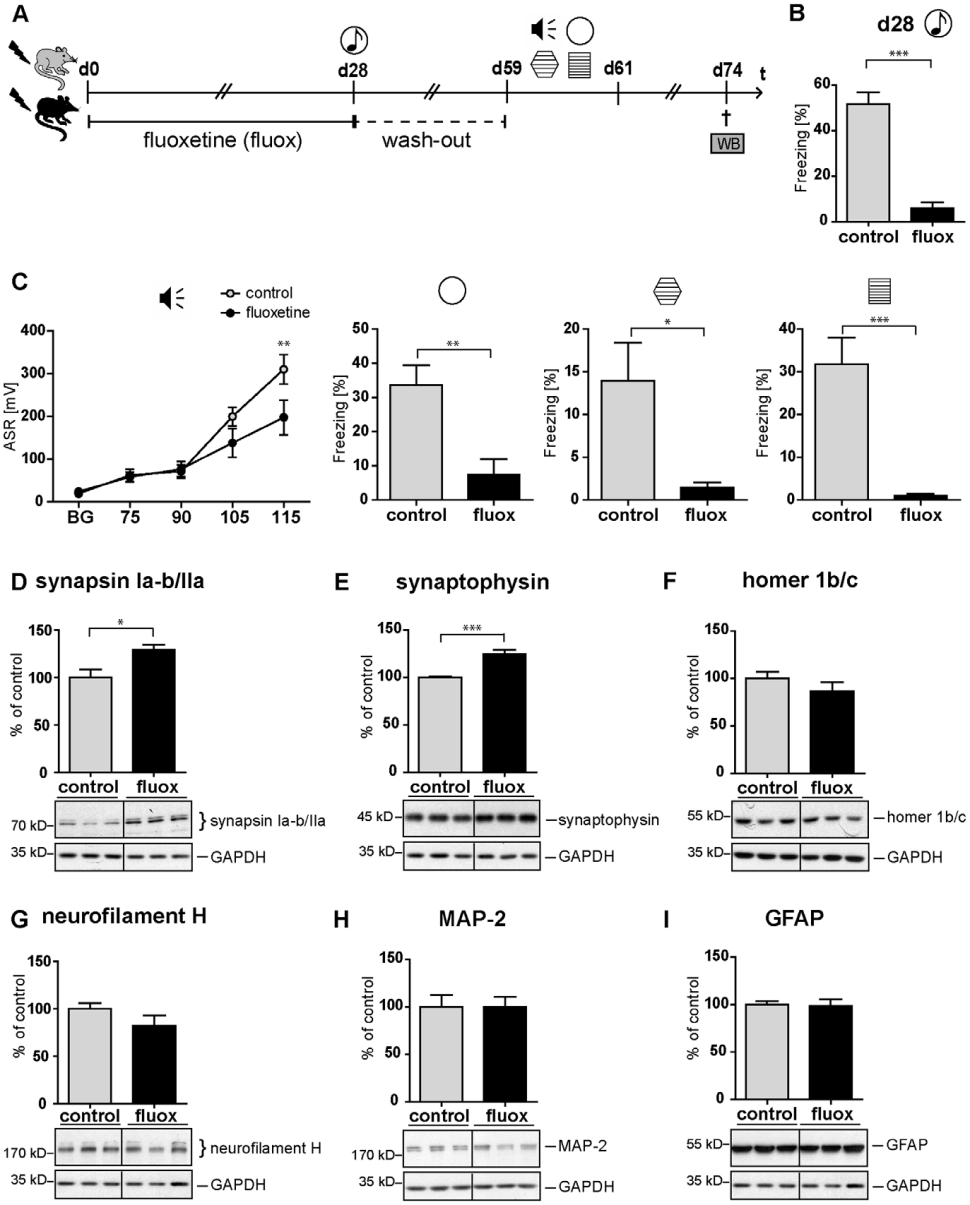
Chronic fluoxetine treatment counteracts both the PTSD-like syndrome and the accompanying hippocampal SV protein reduction. (**A**) Course of experiment: C57BL/6 NCrl mice (batch PTSD IV) were exposed to a single electric footshock and subsequently subjected to treatment with either fluoxetine (20 mg/kg/d) or, for control, drinking water. On day 28, their generalized fear response was assessed (**B**) during the presentation of a neutral tone in the neutral context (t = 7.516, df = 28, p<0.001). Figure S1 provides further details of behavioral protocols. The following 3 days, the dose of fluoxetine was halved (i.e. 10 mg/kg/d) prior to subsequent treatment interruption. After a 4-week washout period, mice were tested on days 59–61 for the intensities of (**C**) their acoustic startle response (F_1,112 fluoxetine_ = 2.072, p = 0.161; F_4,112 INT x fluoxetine = _4.325, p = 0.003), generalized fear response (t (28) = 3.475, p = 0.002), t (28) = 2.607, p = 0.015) and conditioned fear response (t (23) = 4.736, p<0.001). Presented data are means ± SEM, n = 16 (ctrl), n = 14 (fluox). (**D–I**) Representative immunoblots of all candidate proteins and, for control, glycerinaldehyd-3-phosphat-dehydrogenase (GAPDH) analyzed in tissue lysates prepared on day 74 after footshock or control treatment from pooled bilateral hippocampus of footshocked control (ctrl) or footshocked fluoxetine (fluox) treated mice, respectively. Grouped graphical inserts belong to one and the same gel. Graphs show relative expression levels of synapsin Ia–b/IIa (**D**), synaptophysin (**E**), homer 1b/c (**F**), neurofilament (**G**), MAP-2 (**H**), and GFAP (**I**) after normalization to GAPDH. Candidate protein expression levels assessed in fluoxetine-treated mice are presented as percent of those of control mice set at 100% (% of control). Plotted data represent means ± SEM, n = 6. Statistical significance was calculated using students *t*-test and is indicated by * p<0.05, ** p<0.01, *** p<0.001. (synapsin Ia–b/IIa: t (10) = 2.929, p = 0.015; synaptophysin: t (10) = 5.239, p<0.001; homer 1b/c: t (10) = 1.150, p = 0.277; neurofilament H: t (10) = 1.433, p = 0.182; MAP-2: t (10) = 0.007, p = 0.995; GFAP: t (10) = 0.139, p = 0.892).

Both the previously published therapeutic efficacy of chronic fluoxetine treatment in our mouse model of PTSD [30], its reported ability to induce synaptophysin expression in a mouse model of learned helplessness [38], and its reported efficacy in the treatment of PTSD in humans [31], motivated us to analyze whether this SSRI antidepressant is also able to rescue footshock-elicited hippocampal SV protein reduction. For this purpose, we subjected a group of shocked C57BL/6 NCrl mice to either a 4-week fluoxetine (20 mg/kg/d) or control treatment followed by a 4-week wash out period (Figure 6A). This wash-out period allowed for testing specifically the long-lasting (and not the acute) molecular effects of fluoxetine. As expected, fluoxetine treated mice exhibited a marked significant decrease of their acoustic startle and conditioned fear response (day 59 and 61) and a significantly reduced generalized fear response both on day 28 (Figure 6B) and on day 60 after footshock (Figure 6C). The expected relief of behavioral symptoms was accompanied by a significant long lasting (day 74) upregulation of both synapsin Ia–b/IIa and synaptophysin, while the expression of neurofilament H, MAP-2, and GFAP was not significantly altered (Figure 6D–I: synapsin Ia–b/IIa: t (10) = 2.929, p = 0.015; synaptophysin: t (10) = 5.239, p<0.001; homer 1b/c: t (10) = 1.150, p = 0.277; neurofilament H: t (10) = 1.433, p = 0.182; MAP-2: t (10) = 0.007, p = 0.995; GFAP: t (10) = 0.139, p = 0.892).

## Discussion

This study demonstrates for the first time that a loss of hippocampal synaptic proteins is associated with a PTSD-like syndrome in mice. We speculate that this trauma-induced reduction of hippocampal synaptic protein expression levels might contribute to the hippocampal shrinkage we previously found in traumatized mice [11]. This speculation is corroborated by our finding that fluoxetine is also able to counteract both the trauma-induced synaptic protein loss and the behavioral symptoms (Figure 6). Besides fluoxetine [38], [39],other antidepressants, neuroleptics, the anxiolytic neuropeptide S, and mifepristone have also been found to increase synapsin and synaptophysin expression [36], [38], [40]–[43].

The here identified trauma-elicited reduction of hippocampal synaptic proteins (summarized in Table 1) arises either from a decrease of their synthesis rate and/or an increase of their degradation rate. In major depression, that in most cases is accompanied by an increased activity of the hypothalamic pituitary adrenal (HPA) axis, cortisol excess-induced neural apoptosis was proposed to cause hippocampal shrinkage [44]. In contrast, PTSD pathogenesis is predominantly linked to hypoactivation of the HPA axis and thus to hypocortisolism [6]. Against this background, and together with the fact that both hippocampal synapsin and synaptophysin are known to be upregulated by glucocorticoids [45], [46], we speculate that the long-term SV protein downregulation observed in our PTSD mouse model might result from trauma-induced glucocorticoid deficiency.

Furthermore, we assume that in our mouse model neuronal cell death does not grossly contribute to footshock elicited hippocampal shrinkage since we previously found this hippocampal volume loss to be reversible [11]. This assumption leads us to interpret trauma-induced reduction of hippocampal neurofilament H as an indicator of axonal plasticity rather than of neuronal loss. Since also the early stages of certain apoptotic pathways were reported to be reversible [47], we currently cannot fully exclude the possibility that the apoptotic machinery contributes to the posttraumatic hippocampal shrinkage observed here - as it has been already proposed by others, especially in the context of major depression [44]. In addition to the HPA axis, the sympathetic nervous system is also known to contribute to PTSD pathogenesis and hence might also play a role in the induction of posttraumatic long-term synaptic protein loss. We found no reports on catecholamine-mediated regulation of synaptic protein expression, but there are at least some studies reporting norepinephrine to modulate synaptic activity by increasing the phosphorylation status of SV proteins [48].

As far as we know, this study is the first to analyze the long-term effects of acute traumatic stress combined with re-exposure on the expression of neurostructural proteins. In addition to the two presynaptic SV proteins, we show that the postsynaptic protein homer 1b/c is significantly downregulated in the hippocampus of traumatized mice thereby demonstrating the stress responsivity of hippocampal homer 1b/c expression for the first time (Figure 3B). The isoforms homer 1a and homer 2a/b were already known for this property [25], [49]. In addition to synaptic protein loss, we detected a significant reduction of the neuronally expressed neurofilament H on day 2 and a statistical trend in neurofilament H reduction on day 60 after footshock in the hippocampus of traumatized mice. Remarkably, in our PTSD paradigm, on day 28 after application of the traumatic stressor, i.e. in animals not subjected to the stressful behavioral testing, none of the proteins tested was significantly altered in its amount, while on days 2 and 60 the expression levels of all synaptic proteins were significantly reduced (Figure 2 and 3). From that we speculate that stressful behavioral testing on days 28–30 might amplify trauma-elicited synaptic protein loss and axonal retraction. It is well known that repeated stressors intensify the behavioral stress response and hence it is plausible that also the molecular stress response behaves in a similar manner. However, synaptic protein loss sustainably persisted from day 28 to at least day 60 without any in-between application of additional stressors. Since we found expression of the dendritic marker MAP-2 and the astroglial marker GFAP to be unchanged at any time-point and in any brain region tested, we assume that dendritic atrophy or neuronal or glial cell loss do not play a major role in trauma-elicited behavioral symptoms or the accompanying shrinkage of the mouse hippocampus. In addition, our correlation analyses suggest anxiety and hyperarousal symptoms to derive from different molecular pathomechanisms (Figure 5), i.e. we found the hyperarousal symptoms not to correlate with the intensity of SV protein expression in any paradigm tested.

PTSD pathobiology and in particular PTSD associated alterations in fear learning and memory processes are strongly assumed to be linked to synaptic plasticity which hitherto was studied predominantly in the region of the amygdala [50]. Since we concentrated on deciphering the molecular underpinnings of PTSD-associated hippocampal volume loss and due to methodological issues, so far we did not check this important part of the limbic system for microstructural changes, but we intend to address this in future studies. Interestingly, in our PTSD mouse model we found synapsin protein loss in the hippocampus but not the prefrontal cortex which was repeatedly proposed to play a role in the neuropathology of both human and mouse PTSD [51], [52]. From a report demonstrating that the hippocampus is the most vulnerable region during the extremely stressful condition of experimental sepsis [53], we speculate the stress-responsivity of the hippocampus to be higher than that of cortical areas. In accordance with previous reports [33]–[35], our immunohistochemical analysis indicates that synapsin is mainly expressed in the CA3/DG region of mouse hippocampus (Figure 4). Interestingly, PTSD patients were reported to exhibit the most pronounced hippocampal volume loss exactly in this hippocampal subregion [54].

Studies in human twins suggest smaller hippocampal volumes to represent a vulnerability factor for PTSD development rather than a consequence of traumatic stress [55], [56]. In contrast, animal studies propose that stress induces a hippocampal shrinkage [57], [58]. From that, we hypothesize that smaller hippocampal volumes might represent a vulnerability factor for PTSD and that traumatic stress might further diminish the hippocampal volume. This hypothesis is supported by a clinical study showing PTSD duration to negatively correlate with hippocampal volume [59], but additional trials are clearly needed to clarify this hypothesis.

The few studies that have analyzed the molecular underpinnings of hippocampal shrinkage suggested a reduction of hippocampal neurogenesis [60], [61] and gliogenesis as well as enhanced apoptosis [62] and oxidative stress [63] to underlie stress-induced hippocampal volume loss. Despite these efforts, the molecular correlates of hippocampal shrinkage observed in PTSD and other stress related mental disorders [64] still remain elusive. Especially in regard to the repeatedly reported opposite HPA axis pathologies of PTSD and major depression, it even remains unclear whether the hippocampal volume loss accompanying both of these affective disorders derives from identical molecular pathogenic mechanisms.

In summary, this study contributes to the enlightenment of the molecular basis of trauma-induced hippocampal shrinkage. While in a previous study volumetric measurements (*in vivo* Manganese Enhanced Magnetic Resonance Imaging (MEMRI) and *ex vivo* ultramicroscopic analyses) revealed no details on possible structural correlates of hippocampal shrinkage [11], stereological assessment of cell number and neuropil volume as well as ultrastructural microscopic analyses will help to further elucidate the structural and molecular underpinnings of stress-induced hippocampal volume reduction in the future - especially if these investigations will also include human *post mortem* brain specimens.

## Materials and Methods

### Ethics Statement

All experimental procedures were ethically approved by the Committee on Animal Health and Care of Upper Bavaria (Regierung von Oberbayern), Germany (approval ID-AZ: 55.2-1-54-2531-41-09) and were conducted according to the current regulations for animal experimentation in Germany and the European Union (European Communities Council Directive 86/609/EEC).

### PTSD Mouse Model

23 days old male C57BL/6 NCrl mice purchased from Charles River GmbH (Sulzfeld, Germany) were housed in groups of 4 animals for 6 weeks under an inverse 12∶12 h light-dark cycle (lights off: 09∶00 a.m.) with food and water *ad libitum*. Experiments were performed during their activity phase, i.e. between 9∶30 a.m. and 6∶00 p.m., employing our previously published PTSD mouse model [30]: briefly, 28 or 60 (Figure 1) days after a single 1.5 mA electric footshock for 2 s or mock treatment (chamber exposure), intensity of hyperarousal was assessed by evaluating their acoustic startle responses. Their generalized and conditioned fear responses were analyzed by monitoring their freezing behavior upon subsequent exposure to a neutral context, to an experimental context similar to the shock chamber, and to the shock chamber (re-exposure), respectively. Video-taped animal behavior was rated off-line by a trained observer who was blind to the experimental conditions. An overview of detailed behavioral protocols is provided in Figure S1. For immunoblot analyses, groups of mice were sacrificed by cervical dislocation 2 days, 28 days or 60 days after footshock or mock treatment (that means that mice sacrificed on day 60 were killed 30 days after behavioral testing) (Figure 2A) and brain regions were dissected. The results obtained in mice sacrificed on day 60 were validated in a second, independent mouse batch (batch PTSD II, n = 6, Table 2), which was additionally subjected to analysis of avoidance behavior (CODA, Figure S1, S2) as described in [65]. The CODA test is *not* considered to be stressful as mice are *not* coercively exposed to trauma-related cues but are placed in a compartment with home cage material and are free to enter a compartment with a smell reminding of the shock context or to enter another compartment with a neutral smell. Expression data of obtained from mice sacrificed on day-60 (batch PTSD I and II, Table 2) was fused, except for synaptophysin which was analyzed only in batch PTSD I. An overview of the four batches of mice employed for this study is shown in Table 2.

**Table 2:** Overview of mouse batches employed.

Batch Name	Analyses	Batch Size(n = number of mice per group)
PTSD I	Behavior (d28–30) + WB (d2, d28, d60)	Behavioral tests: n = 16 per time point; WB: n = 6 per time point
PTSD II (confirmatory batch)	Behavior (d28–30) + WB (d60) + IHC (d60)	WB: n = 6; IHC: n = 6
PTSD III	Behavior (d59–60)	n = 14 (no shock), n = 16 (shock)
PTSD IV (fluoxetine treatment)	Behavior (d59–60) + WB (d74)	Behavioral tests: n = 16 (ctrl), n = 14 (fluoxetine); WB: n = 6

Abbreviations: posttraumatic stress disorder (PTSD); western blot (WB); behavioral analysis (Behavior). For detailed explanation of the PTSD stress paradigms and the course of pharmacological fluoxetine treatment see methods and results chapter.

### Fluoxetine Treatment

A group of 30 male C57BL/6 NCrl mice aged 10 weeks (batch PTSD IV, Figure 6A, and Table 2) was exposed to a single inescapable footshock as described above. The treatment group (n = 14) was subjected to chronic fluoxetine (Ratiopharm, Ulm, Germany) treatment, which was administered in a dose of 20 mg/kg per day for 4 weeks via drinking water as we described before in [30], while the control group (n = 16) received drug-free drinking water. On day 28 after shock, fluoxetine efficacy was assessed by evaluating the generalized fear response for 60 s during the presentation of a neutral tone (80 dB, 9 kHz) in the neutral context (for detailed behavioral protocols see Figure S1). After assessment of fluoxetine efficacy the fluoxetine dose was halved (10 mg/kg per day) for 3 days and a 4 week washout period during which mice received pure drinking water. On days 59 to 61, animals were tested for PTSD-like symptoms as described above and depicted in Figure 6A. On day 74, mice were sacrificed by cervical dislocation and bilateral hippocampi were prepared for immunoblot analyses (n = 6).

### Western Blot (WB) Analysis

Tissues were lysed by homogenization and sonification. Equal amounts (10 µg) of total protein were separated by SDS-PAGE and transferred to nitrocellulose membranes (Millipore, Billerica, USA). After blocking with 5% non-fat milk in Tris-buffered saline with 0.1% Tween-20 (TBS/T), membranes were incubated overnight at 4°C with the respective primary antibodies: polyclonal rabbit anti-synapsin antibody (diluted 1∶1000, Synaptic Systems, Göttingen, Germany) or rabbit polyclonal anti-synaptophysin antibody (diluted 1∶2000, Santa Cruz Biotechnology, Santa Cruz, CA, USA) or rabbit polyclonal anti-homer 1b/c antibody (diluted 1∶1000, Santa Cruz Biotechnology, Santa Cruz, CA, USA) or mouse monoclonal anti-neurofilament antibody (diluted 1∶1000, Abcam, Cambridge, UK) or mouse monoclonal MAP-2 antibody (diluted 1∶1000, SIGMA, St. Louis, MO, USA) or rabbit polyclonal GFAP antibody (diluted 1∶1000, Dako, Glostrup, Denmark) and with mouse monoclonal anti-GAPDH antibody (diluted 1∶2000, Santa Cruz Biotechnology, Santa Cruz, CA, USA). After washing, membranes were incubated with horseradish peroxidase (HRP)-conjugated secondary antibodies (goat anti-rabbit, diluted 1∶30000, SIGMA, St. Louis, MO, USA or goat anti-mouse, diluted 1∶50000, SIGMA, St. Louis, MO, USA). Signals were visualized on radiographic films (Bender, Baden-Baden, Germany) after incubating blots with enhanced chemoluminescence (ECL) developing solution (Millipore, Billerica, USA). Expression levels of all candidate proteins were normalized to glycerinaldehyde-3-phosphate dehydrogenase (GAPDH) signals. Densitometric analyses were carried out using ImageJ Software (Rasband, W.S., ImageJ, U.S. National Institutes of Health Bethesda, Maryland, USA, http://rsb.info.nih.gov/ij/, 1997–2009). Each western blot experiment was performed in at least three technical replicates.

### Immunohistochemistry (IHC)

On day 60 after footshock, isoflurane-anesthetized mice (n = 6, batch PTSD II; see Table 2) were perfused transcardially with phosphate-buffered saline (PBS) followed by 4% paraformaldehyde (PFA) for 15 min using a peristaltic pump. Total brains were dissected and kept in 4% PFA overnight, followed by one week in 30% sucrose at 4°C. Coronal sections of shock frozen murine brains (40 µm) were kept in storage medium (25% glycerol, 25% ethylenglycol in PBS) at −20°C. Then, sections were washed with PBS, blocked with blocking solution (10% bovine serum albumin (BSA) and 1% Triton X-100 in PBS) and incubated overnight at room temperature with anti-synapsin (Synaptic Systems, Göttingen, Germany) diluted 1∶1000 in BSA-buffer solution (1% BSA and 0.3% Triton X-100 in PBS). The next day, after washing, sections were incubated for 2 h at room temperature with the fluorophore-conjugated secondary antibody (donkey anti-rabbit alexa594, Invitrogen, Paisley, UK) diluted 1∶300 in BSA-buffer solution. Subsequently, sections were washed with PBS including 0.2 mg/ml of the nuclear stain DAPI (4′,6-diamidino-2-phenylindole) in one washing step. Images were obtained with an Olympus IX81 confocal microscope (Olympus, Hamburg, Germany).

### Statistical Analysis

The conditioned and generalized fear responses were statistically analyzed employing a two-tailed student’s *t*-test. Statistical analysis of acoustic startle response data was performed by two-way repeated measures ANOVA and Bonferroni post-hoc tests. Western blot data were analyzed by performing a two-tailed student’s *t*-test. Statistical significance is ascribed at p<0.1 as a statistical trend (t); * p<0.05; ** p<0.01; *** p<0.001.

Correlation studies were performed according to the procedure published by Wu et al. (2007) and Zink et al. (2010) [36], [37] employing GraphPad Prism 5 (GraphPad Software, La Jolla, CA, USA): by calculating the Pearson correlation coefficient we correlated the mean relative synapsin Ia–b/IIa, synaptophysin, or homer 1b/c protein expression values (means from three replicates) of every mouse sacrificed on day 60 after shock, i.e. of shocked and non-shocked mice belonging to batches PTSD I and II, with the numeric values representing the intensities of either its conditioned fear, generalized fear, or acoustic startle response on day 28–30 after shock or mock treatment (Figures 5A–C).

## Supporting Information

Figure S1 **Protocols of behavioral tests**. The different phases of the behavioral test protocols are indicated in Roman numerals. Mouse behavior was scored in the phase highlighted in grey. At day 0 a 1.5 mA single electric footshock was applied as depicted in (B). After 198 s in the shock chamber, the footshock was administered for 2 s, followed by additional 60 s in the shock chamber. Control mice remained in the shock chamber for 260 s without administration of the electric shock. The acoustic startle response was assessed as described in detail in Golub *et al* (2011) in batches PTSD I (Figure 1B), PTSD II (data not shown), PTSD III (Figure 1G) and PTSD IV (Figure 6C). Briefly, after 300 s of habituation in the startle chamber, 16 control trials and 30 startle stimuli of 20 ms duration of each intensity (INT), 75, 90, 105, and 115 dB, were presented in a pseudorandom order with an interstimulus interval of 15 s. For analysis of generalized fear (Siegmund and Wotjak, 2007), mice of batches PTSD I (Figure 1C), PTSD II (data not shown), PTSD III (Figure 1H) and PTSD IV (Figure 6C) were exposed to a neutral context for 180 s. Then a neutral tone (80 dB, 9 kHz) was presented for 180 s and mice remained for additional 60 s in the neutral context before returning to their home cage. As described in detail in Siegmund and Wotjak (2007), generalized fear was further tested in a context with the grid as a dominant reminder of the shock context in batches PTSD I (Figure 1D), PTSD II (data not shown), PTSD III (Figure 1I) and PTSD IV (Figure 6C) and conditioned fear was assessed by scoring the freezing response for 180 s in the shock context in batches PTSD I (Figure 1E), PTSD II (data not shown), PTSD III (Figure 1J), and PTSD IV (Figure 6C). In the PTSD II batch mice were additionally tested in a conditioned odor avoidance task (CODA) as described in Pamplona *et al.* (2011) (Figure S2). Briefly, mice were placed in a box with three compartments: a central compartment containing home-cage nesting material. This compartment is flanked by compartments scented with either 70% ethanol or 1% acetate. After 300 s in the central compartment (habituation phase), mice had access to all three compartments for additional 300 s (test phase) and the time spent in each compartment was scored. To test for treatment efficacy of the fluoxetine treatment, after 180 s in the neutral context, the freezing response was assessed during a 60 s presentation of a neutral tone in a neutral context. After additional 60 s in the neutral context, mice returned to their home cage.
(PDF)

Figure S2 **Footshocked mice show a generalized avoidance behavior.** On day 42 after footshock conditioned odor avoidance (CODA) was tested in a chamber with three compartments: the ethanol-scented compartment, reminding of the shock context, the nest compartment with home-cage material in the center, and a third compartment with a neutral, acetate, odor. The time spent in the ethanol, the nest, and the acetate compartments, and, in addition, the latency to the first compartment entry are depicted in the graph. Data are represented as means ± SEM, n = 16 (batch PTSD II). Statistical analysis was performed using Student‘s t-test and is indicated by t p<0.1, ** p<0.01, *** p<0.001.
(PDF)
