# Supplementary figures and images for: Long-Lasting Hippocampal Synaptic Protein Loss in a Mouse Model of Posttraumatic Stress Disorder
Source: PLoS One. 2012 Aug 10;7(8):e42603. doi: 10.1371/journal.pone.0042603 (PMC3416820; doi:10.1371/journal.pone.0042603)

**Figure S1**

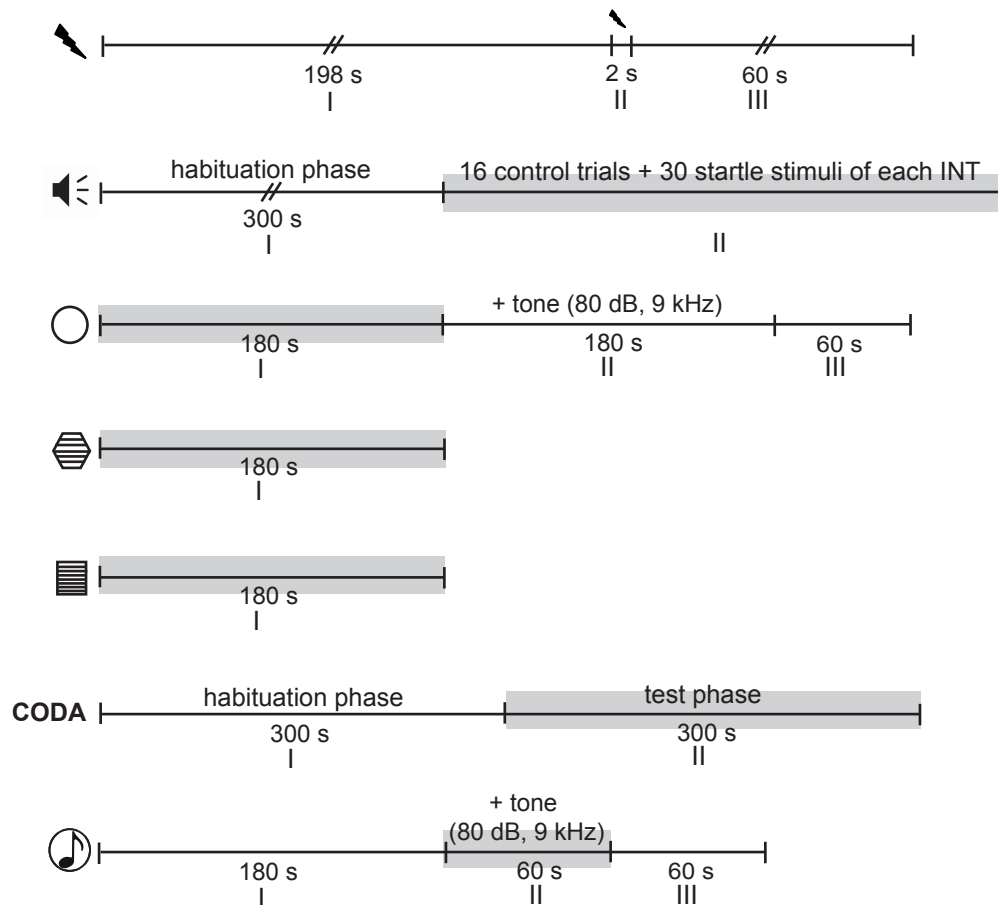

Supplement: Figure S1 — Protocols of behavioral tests. The different phases of the behavioral test protocols are indicated in Roman numerals. Mouse behavior was scored in the phase highlighted in grey. At day 0 a 1.5 mA single electric footshock was applied as depicted in (B). After 198 s in the shock chamber, the footshock was administered for 2 s, followed by additional 60 s in the shock chamber. Control mice remained in the shock chamber for 260 s without administration of the electric shock. The acoustic startle response was assessed as described in detail in Golub et al (2011) in batches PTSD I (Figure 1B), PTSD II (data not shown), PTSD III (Figure 1G) and PTSD IV (Figure 6C). Briefly, after 300 s of habituation in the startle chamber, 16 control trials and 30 startle stimuli of 20 ms duration of each intensity (INT), 75, 90, 105, and 115 dB, were presented in a pseudorandom order with an interstimulus interval of 15 s. For analysis of generalized fear (Siegmund and Wotjak, 2007), mice of batches PTSD I (Figure 1C), PTSD II (data not shown), PTSD III (Figure 1H) and PTSD IV (Figure 6C) were exposed to a neutral context for 180 s. Then a neutral tone (80 dB, 9 kHz) was presented for 180 s and mice remained for additional 60 s in the neutral context before returning to their home cage. As described in detail in Siegmund and Wotjak (2007), generalized fear was further tested in a context with the grid as a dominant reminder of the shock context in batches PTSD I (Figure 1D), PTSD II (data not shown), PTSD III (Figure 1I) and PTSD IV (Figure 6C) and conditioned fear was assessed by scoring the freezing response for 180 s in the shock context in batches PTSD I (Figure 1E), PTSD II (data not shown), PTSD III (Figure 1J), and PTSD IV (Figure 6C). In the PTSD II batch mice were additionally tested in a conditioned odor avoidance task (CODA) as described in Pamplona et al. (2011) (Figure S2). Briefly, mice were placed in a box with three compartments: a central compartment containing home-cag [file pone.0042603.s001.pdf]

Figure S2

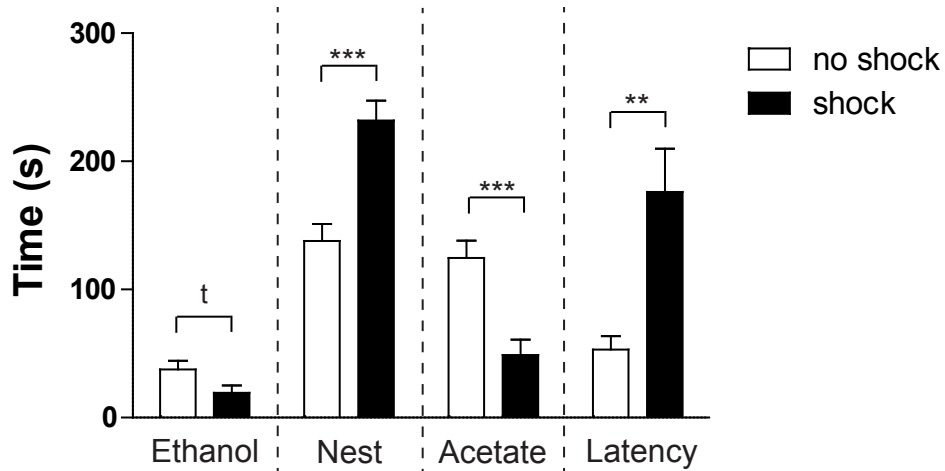

Supplement: Figure S2 — Footshocked mice show a generalized avoidance behavior. On day 42 after footshock conditioned odor avoidance (CODA) was tested in a chamber with three compartments: the ethanol-scented compartment, reminding of the shock context, the nest compartment with home-cage material in the center, and a third compartment with a neutral, acetate, odor. The time spent in the ethanol, the nest, and the acetate compartments, and, in addition, the latency to the first compartment entry are depicted in the graph. Data are represented as means ± SEM, n = 16 (batch PTSD II). Statistical analysis was performed using Student‘s t-test and is indicated by t p<0.1, ** p<0.01, *** p<0.001. (PDF) [file pone.0042603.s002.pdf]
